# Quantitative validation of nicotine production in tea (*Camellia sinensis* L.)

**DOI:** 10.1371/journal.pone.0195422

**Published:** 2018-04-09

**Authors:** Takashi Ikka, Hiroto Yamashita, Ikuya Kurita, Yasuno Tanaka, Fumiya Taniguchi, Akiko Ogino, Kazuya Takeda, Nobuhiro Horie, Hiroshi Hojo, Fumio Nanjo, Akio Morita

**Affiliations:** 1 Faculty of Agriculture, Shizuoka University, Shizuoka, Shizuoka, Japan; 2 Tea Research Division of NARO Institute of Fruit and Tea Science (NIFTS), Makurazaki, Kagoshima, Japan; 3 Mitsui Norin Co., Ltd., Fujieda, Shizuoka, Japan; Henan Agricultural University, CHINA

## Abstract

Endogenous nicotine was confirmed to be present in tea plants (*Camellia sinensis* L.) by liquid chromatography-tandem mass spectrometry of tea samples from tea-producing regions in six Asian countries. All samples contained nicotine (0.011–0.694 μg g^−1^ dry weight). Nicotine contents remained constant during manufacturing of green, oolong and black teas, implying that nicotine is stable against heating, drying, enzymatic oxidation and mechanical damage during processing. Flower buds and seeds of cultivar Yabukita also contained nicotine (0.030–0.041 μg g^−1^ dry weight). A comparison of two cultivars revealed that higher nicotine contents were found in the black tea cultivar Benifuki. All plant parts of hydroponic Yabukita contained nicotine (0.003–0.013 μg g^−1^ dry weight). Tea cells cultured in B5 medium as well as roots and stems of tea seedlings contained nicotine levels similar to those of new leaves from field-grown plants. Although the levels of endogenous nicotine in tea plants are extremely low and sample contamination cannot be discounted, these levels exceed the maximum acceptable limit in Japan (0.01 μg g^−1^ dry weight).

## Introduction

Tea, derived from the tea plant (*Camellia sinensis* L.), is one of the world’s most popular beverages. Various types, such as green, black and/or oolong, are consumed in different localities. Unlike oolong and black teas, green tea is made without the use of withering and enzymatic oxidation (e.g. polyphenol oxidase and peroxidase) processes. Consequently, catechins and ascorbic acid contents of green tea are generally higher than those of other teas [1, 2]. Green tea, produced mainly in China, Vietnam and Japan, is classified into Chinese and Japanese styles [3]. Japanese-style green tea typically differs from Chinese in the way in which the leaves are heated: Japanese-style tea leaves are heated with a steaming machine—which minimizes the deactivation of oxidation enzymes—whereas Chinese tea production involves a parching machine (S1 Fig). Sencha, the most common Japanese green tea, plays an important role in Japanese culture. The export volume of Japanese green tea has recently increased greatly. Because these compounds are beneficial to human health, their higher levels in green tea have resulted an increase in green tea production and consumption—up to one-third that of black tea.

Nicotine, which is an alkaloid compound mainly found in the genus Nicotiana (e.g. tobacco), has been detected in many food crops [4]. Nicotine acts as an agonist at nicotinic acetylcholine receptors, therefore, it has been used as a pesticide because of its toxicity to organisms. Previous studies have revealed the presence of nicotine not only in solanaceous crops, namely, potato (*Solanum tuberosum*), tomato (*S*. *lycopersicum*), eggplant (*S*. *melongena*) and pepper (*Capsicum annum*), but also in wild mushrooms and cauliflower (*Brassica oleracea*) [5–11]. These findings suggest that nicotine is widely distributed in plants and fungi. In a survey reported by the European Food Safety Authority (EFSA), nicotine concentrations were measured in 332 tea samples (87 green, 239 black and 6 Chinese white) originating from numerous tea-producing countries [4]. Nicotine was detected in 274 samples, with contents of 53 samples found to be over the default maximum residue level (MRL) of 0.01 mg kg^−1^ dry weight (DW). Prior to this EFSA report, other several studies uncovered nicotine in tea at levels ranging from 0 to 28.01 mg kg^−1^ DW [5–8]. In Japan, where no nicotine-containing pesticides have been listed in the Agricultural Chemicals Regulation Law since 2001, the MRL of nicotine is set at 0.01 mg kg^−1^ DW. Japanese green tea should thus have no detectable exogenous nicotine. The nicotine content of Japanese green tea was not measured in any of the above-mentioned studies.

Tobacco (*Nicotiana tabacum*) is the only plant in which nicotine biosynthesis has actually been confirmed to occur [6]. Various authors [5, 6] have proposed that the nicotine detected in tea is due to insecticide contamination. Sheen [5] has suggested that the content of nicotine in tomato is lessened during processing due to enzymatic oxidation and other chemical reactions. In contrast, however, a screening for nicotine in various processed tomato products by Siegmund et al. [8] has indicated that nicotine is thermally stable and does not degrade or evaporate during processing. In tobacco leaves, various post-harvest reactions during curing degrade nicotine into its nitrogen oxide as well as into cotinine and other alkaloids [12]. Also in tomato, Siegmund et al. [8] has observed that nicotine contents in tomato decrease with increasing ripeness and differ between varieties. Taken together, these findings suggest that several factors affect nicotine content in plants. Nevertheless, the above-mentioned assumptions as well as the possibility of nicotine biosynthesis have not been confirmed in plants other than tobacco.

To determine whether endogenous nicotine is present in Japanese green tea, in this study we measured nicotine contents of several types of tea from Japan and five other Asian countries. Nicotine was detected in all analyzed Japanese green tea samples, but the contents were lower than those of black teas. To determine the effect of different manufacturing processes used to generate teas such as green, oolong and black, we next measured nicotine contents using two Japanese cultivars: Yabukita, an important Japanese green tea cultivar, and Benifuki, a newly introduced black tea cultivar and Assam hybrid. We also investigated the nicotine contents of various tea plant organs (leaves, stems, roots, flower buds and seeds) and explored seasonal changes in nicotine contents of field-grown tea plants. The nicotine content of aseptic cultured tea cells was also determined. Finally, the origin of nicotine in tea was considered in the light of these findings.

## Materials and methods

### Plant materials

#### Tea from various tea-producing districts in Asia

Thirty-two green tea (*C*. *sinensis* var. *assamica*), 79 black tea (*C*. *sinensis* var. *sinensis* and/or *C*. *sinensis* var. *assamica*) and 1 oolong tea (*C*. *sinensis* var. *sinensis*) sample(s) were subjected to nicotine analysis (S1 Table). The Indian tea samples were purchased from 16 and 3 tea estates in Darjeeling and Assam, respectively, by Mitsui Norin Co. Ltd., one of the major tea companies in Japan. With the exception of one estate in Assam from which no autumn sample was obtained, tea collections were made during all four crop seasons (first, second, rain/monsoon and autumn; S4 Fig). All samples were from organically grown tea leaves received Japanese Agricultural Standard (JAS) certification, i.e. grown without the use of chemicals including insecticides. In the case of the Japanese samples, which were collected by the Tea Research Division of the NARO Institute of Fruit and Tea Science (NIFTS) (Makurazaki, Kagoshima, Japan), the leaves came from a cultivated tea garden where no insecticides (including nicotine) have been used for several decades. Other tea samples were purchased from organic cultivation in local markets of each country. All green teas collected in Japan were typical Japanese types (Sencha), produced using steam, while those from Taiwan were Chinese types (pan-fired). Tea samples were dried in a freeze dryer (FDU-2000, EYELA, Tokyo, Japan) and then ground into a fine powder with a vibrating mill (SA300, Yamato, Tokyo, Japan). The samples were stored in desiccators until nicotine analysis.

#### Tea subjected to different manufacturing processes

New shoots of 30-year-old plants of two tea cultivars (Yabukita and Benifuki) were harvested during the first crop season on 25 April 2015 at the NIFTS tea garden (Makurazaki, Kagoshima, Japan). The shoots were immediately used to manufacture Japanese (Sencha, steaming type) and Chinese (pan-fired tea, panning type) green teas, oolong tea and black teas following the processes shown in S1 Fig. Following processing, samples were collected for nicotine analysis and stored in a freezer at −20°C until tea manufacturing. The manufactured tea samples were then freeze-dried, ground and stored in the same manner as described above.

#### Tea from each growing season

New leaves, new stems, mature leaves and roots of Yabukita and Benifuki were harvested from the NIFTS tea garden (Makurazaki, Kagoshima, Japan) on 18 April (first crop season), 18 June (second crop season) and 13 August (third crop season) in 2014. Mature leaves and roots were also harvested on 27 February during the off season in 2015. The roots were collected from the soil layer between hedges at a depth of 10 cm. After washing three times with deionized water, the samples were freeze-dried, ground and stored in the same manner as described above.

#### Tea plant parts and cultured tea cells

One-year-old rooted cuttings of cultivars Yabukita and Benifuki were transplanted on 8 April 2014 into Wagner pots (1/2,000a) containing 10 L of aerated nutrient solution and grown in a greenhouse at Shizuoka University (Shizuoka, Shizuoka, Japan). The nutrient solution, prepared following the method of Konishi et al. [13], was renewed weekly. The pH of the hydroponic solution was adjusted to 4.2 with 5 N H_2_SO_4_. After 90 days (on 7 July 2014), the tea plants were harvested and divided into six parts: new leaves, new stems, mature leaves, mature stems, lignifying roots and white roots (S2 Fig).

Flower buds of 35-year-old Yabukita tea plants were collected from the tea garden of the Shizuoka Prefecture Tea Research Center (Kikugawa, Shizuoka, Japan) on 5 November 2015. The flower buds were immediately divided into petals, filaments and anthers (Panel A in S3 Fig). On 26 October 2015, fully mature fruits containing one to four seeds were collected from 20-year-old Yabukita tea plants in a private tea garden in Shizuoka, Japan. After peeling away the pericarp, the outer seed coat was broken with a hammer, and the outer and inner seed coats were carefully removed. The collected flower buds and tea seeds were freeze-dried, ground and stored as described earlier. Because collection of adequate samples for nicotine analysis was difficult, only a single replicate was performed.

Suspension-cultured tea cells (Panel B in S3 Fig) were prepared from calli derived from anthers of Yabukita [14]. The cells were subcultured weekly in B5 medium at pH 5.6 in darkness at 25°C on a rotary shaker (100 rpm). Exponentially growing cells were harvested after 9 days by suction filtration, suspended and rinsed three times with a simple solution containing 0.2 mM CaCl_2_ at pH 5.6. The cells were frozen in liquid nitrogen and freeze-dried. B5 medium was also collected before and after 9 days cultivation and stored at −20°C until analysis. The samples were freeze-dried, ground and stored in the same manner as described above.

Aseptic tea seedlings were prepared following the method of Morita et al. [15] from the same tea seeds as mentioned above. Four months after germination, the aseptic tea seedlings were divided into leaves, stems and roots (Panel C in S3 Fig) and freeze-dried, ground and stored as described above.

### Measurement of nicotine

Nicotine extraction from tea samples was carried out according to the modified QuEChERS method of Cavalieri et al. [16]. One gram of powdered tea sample was added to a 50-mL polytetrafluoroethylene (PTFE) centrifuge tube along with 50 μL of nicotine d_3_ internal standard (Sigma, MO, USA) at a concentration of 5 μg mL^−1^. To this mixture was added 20 mL of 0.1 M ammonia solution followed by incubation for 10 min to allow for swelling of the tea powder. After adding 10 mL acetonitrile, 6 g MgSO_4_ and 1.5 g CH_3_COONa, the solution was mixed on a shaker at 3,000 rpm for 5 min (SA300, Yamoto, Tokyo, Japan). Following centrifugation at 5,000 ×*g* for 4 min, 1.5 mL of the resulting supernatant was transferred to a 2-mL PTFE centrifuge tube containing 50 mg of Primary Secondary Amine (Agilent, CA, USA) and 150 mg of MgSO_4_. The sample was mixed for 1 min and then centrifuged at 18,000 ×*g* for 5 min. The supernatant was analyzed by liquid chromatography-tandem mass spectrometry (LC-MS/MS). Nicotine in B5 medium from cultured tea cells was also extracted using the same procedure, except that the final concentration of added ammonia solution was 0.1 M.

Nicotine was quantified on a liquid chromatograph (1100 series, Agilent, CA, USA) equipped with a triple quadrupole mass spectrometer (3200 QTRAP®, Sciex, MA, USA) with a TurboIonSpray® (Sciex, MA, USA) electrospray ionization source operated in positive ionization mode. The chromatographic analysis was performed on a 150 × 2.1 mm InertSustain phenylhexyl column (GL Science, Tokyo, Japan). Acetonitrile (B) and 20 mM ammonium hydrogen carbonate (A) were used as the mobile phase at a flow rate of 0.25 mL min^−1^. Elution was performed with the following gradient: initial concentration of 10% B, followed by a 0.1-min linear gradient from 10% to 35% B, a 0.9-min hold at 35% B, a 0.1-min linear gradient from 35% to 50% B, a 1.9-min hold at 50% B, a 2.0-min linear gradient from 50% to 75% B, a 2.0-min hold at 75% B, a 0.1-min gradient from 75% to 10% B, and a final concentration of 10% B for 4.9 min.

Mass spectrometry parameters were optimized as follows: ion spray voltage (5500 V); ionization source temperature (600°C); curtain and CAD gases (138 kPa); gas 1 (552 kPa); gas 2 (483 kPa). Data analyses were performed using Analyst software (version 1.6, Sciex, MA, USA). Chemicals used for nicotine determination were LC/MS grade or analytical grade. The limit of quantification (LOQ) of our method was set at 0.005 μg g^−1^ DW, the minimum concentration giving a signal-to-noise ratio of at least 10.

### Statistical analysis

Data were analyzed by Tukey’s and Dunnett’s tests. A *P*-value < 0.05 was considered to represent a significant difference.

## Results

### Analysis of tea collected from different tea-producing districts in Asia

Nicotine contents of tea samples collected from different tea-producing districts in Asia are shown in Table 1. All samples had nicotine contents above 0.01 μg g^−1^ DW. The maximum detected nicotine content, 0.694 μg g^−1^ DW, was in black tea from a Darjeeling organic tea estate; the minimum, 0.011 μg g^−1^ DW, was found in green tea from Taiwan (Table 1). The average nicotine content of Japanese green tea, 0.019 μg g^−1^ DW (0.013–0.040 μg g^−1^ DW), was lower than that of Assam (0.331 μg g^−1^ DW) and Darjeeling (0.273 μg g^−1^ DW). With respect to tea types, the average nicotine content of green tea (0.019 μg g^−1^ DW) was significantly lower than that of black tea (0.274 μg g^−1^ DW).

**Table 1:** Nicotine contents of tea samples obtained from tea-producing areas in Asia.

Tea Samples					Nicotine content (μg g^−1^ dry weight)					
Type	Subtype	Country or district		Number	Average	±	S.D.	Minimum	Median	Maximum
Green tea	Sencha	Japan		31	0.019	±	0.006	0.013	0.017	0.040
	Pan-fired	Taiwan		1	0.011					
	Sub total			32	0.019	±	0.006	0.011	0.017	0.040
Black tea		India	Assam	11	0.331	±	0.069	0.246	0.332	0.449
			Darjeeling	64	0.273	±	0.151	0.100	0.219	0.694
		China		1	0.289					
		Taiwan		1	0.121					
		Indonesia		1	0.101					
		Vietnam		1	0.024					
	Sub total			79	0.274	±	0.145*	0.024	0.245	0.694
Oolong tea		Taiwan		1	0.020					
**Total**				**112**	**0.199**	**±**	**0.169**	**0.011**	**0.189**	**0.694**

* Significant difference between green tea and black tea (t-test, *P* < 0.01).

Seasonal fluctuation in nicotine contents of Indian black tea collected from 19 tea estates is shown in S4 Fig. Because we observed a different pattern of fluctuation on each tea estate, no overall trend was discernable across estates.

### Analysis of nicotine content as a function of manufacturing process

Nicotine contents of tea derived from green tea (Sencha and pan-fired), oolong tea and black tea manufacturing processing are shown in Table 2. After plucking, fresh tea leaves containing nicotine contents of 0.021 μg g^−1^ DW (in Yabukita) and 0.026 μg g^−1^ DW (in Benifuki) were used for the manufacturing treatments. Although heating temperature, treatment period and the application of fermentation and/or withering (oxidation by oxidative enzymes in leaves) varied among processes, nicotine levels in processed tea leaves were unchanged relative to fresh leaves regardless of the manufacturing process used.

**Table 2:** Nicotine contents of tea leaves following green (Sencha and pan-fired), oolong and black tea manufacturing processes.

Process	Nicotine content (μg g^−1^ dry weight)					
	Yabukita			Benifuki		
Fresh leaves	0.021	±	0.002	0.026	±	0.005
Green tea (Sencha)						
Steaming	0.021	±	0.001	0.023	±	0.001
Primary drying	0.020	±	0.002	0.025	±	0.004
Rolling	0.020	±	0.001	0.022	±	0.001
2nd drying	0.017	±	0.001	0.022	±	0.001
Final rolling	0.018	±	0.001	0.023	±	0.002
Drying	0.020	±	0.003	0.025	±	0.001
Green tea (Pan-fired)						
1st panning	0.021	±	0.005	0.021	±	0.001
1st rolling	0.018	±	0.001	0.023	±	0.001
2nd panning	0.019	±	0.001	0.024	±	0.000
2nd rolling	0.021	±	0.000	0.023	±	0.002
2nd drying	0.018	±	0.002	0.022	±	0.001
Drying	0.021	±	0.002	0.023	±	0.002
Oolong tea						
Sunlight withering	0.020	±	0.001	0.024	±	0.001
Indoor withering	0.018	±	0.001	0.024	±	0.002
Panning	0.021	±	0.002	0.024	±	0.001
Rolling	0.018	±	0.002	0.026	±	0.002
2nd rolling	0.020	±	0.000	0.025	±	0.002
Drying	0.019	±	0.001	0.026	±	0.002
Black tea						
Indoor withering	0.020	±	0.002	0.028	±	0.000
Rolling	0.019	±	0.002	0.028	±	0.001
Fermentation	0.019	±	0.002	0.027	±	0.003
Drying	0.021	±	0.001	0.025	±	0.001

Values are means±S.D. (*n* = 3).

### Analysis of nicotine content according to growing season

Table 3 displays seasonal changes in nicotine contents of new leaves, new stems, mature leaves and roots of Yabukita and Benifuki. In new leaves and stems of Yabukita, nicotine contents during the first crop season (0.010 and 0.011 μg g^−1^ DW, respectively) were significantly lower than those during the second (0.023 and 0.033 μg g^−1^ DW) and third (0.022 and 0.036 μg g^−1^ DW). Nicotine contents in these two organs were highest in Benifuki during the third season (0.036 and 0.032 μg g^−1^ DW). Although nicotine contents in mature leaves of both cultivars were highest in the off season (0.024 μg g^−1^ DW in Yabukita and 0.021 μg g^−1^ DW in Benifuki), the ranges of their seasonal changes were smaller than those of new leaves and new stems. In roots of both cultivars, nicotine contents remained constant (ca. 0.028 μg g^−1^ DW in Yabukita and 0.030 μg g^−1^ DW in Benifuki). When averaged over first to third crop seasons, the highest nicotine contents were found in roots.

**Table 3:** Nicotine contents of new leaves, new stems, mature leaves and roots of two tea cultivars (Yabukita and Benifuki) harvested during first, second and third crop seasons and the off season in 2014 and 2015.

Parts	Cultivars	Crop season (nicotine content, μg g^−1^ dry weight)				Statistical analysis*		
		1st season	2nd season	3rd season	Off- season	Cultivar	Season	Cultivar × Season
		(18th Apr.)	(18th Jun.)	(13th Aug.)	(27th Feb.)			
New leaves	Yabukita	0.010 ± 0.001 a	0.023 ± 0.003 b	0.022 ± 0.004 b	n.t.	*P* < 0.05	*P* < 0.01	*P* < 0.05
	Benifuki	0.015 ± 0.004 a	0.016 ± 0.001 a	0.036 ± 0.007 b	n.t.			
New stems	Yabukita	0.011 ± 0.000 a	0.033 ± 0.005 b	0.036 ± 0.008 b	n.t.	NS	*P* < 0.01	NS
	Benifuki	0.013 ± 0.000 a	0.023 ± 0.001 a	0.032 ± 0.003 b	n.t.			
Mature leaves	Yabukita	0.018 ± 0.002 ab	0.014 ± 0.001 a	0.013 ± 0.000 a	0.023 ± 0.003 b	NS	*P* < 0.01	NS
	Benifuki	0.019 ± 0.004 ab	0.014 ± 0.001 a	0.015 ± 0.001 ab	0.021 ± 0.001 b			
Roots	Yabukita	0.028 ± 0.001	0.027 ± 0.001	0.027 ± 0.001	0.028 ± 0.001	*P* < 0.01	NS	NS
	Benifuki	0.028 ± 0.002	0.030 ± 0.003	0.031 ± 0.001	0.031 ± 0.001			

Data represent means ± S.D. (*n* = 3). n.t.: not tested. Different letters within the same row indicate significant differences (Tukey’s HSD test, *P* < 0.05).

* Significant differences among organs of the two cultivars were estimated by two-way ANOVA. NS: not significant (*P* > 0.05).

### Analysis of nicotine in various plant organs and cultured tea cells

Nicotine contents in Yabukita and Benifuki cultured hydroponically for 90 days are given in Table 4. In Yabukita, the lowest nicotine contents were in new shoots (0.003 μg g^−1^ DW, below the LOQ), with contents similar among other plant parts (0.009–0.013 μg g^−1^ DW). In Benifuki, in contrast, white roots and lignifying roots contained nicotine at a significantly higher level than the contents found in leaves and stems.

**Table 4:** Nicotine contents of various plant organs of two hydroponically cultivated tea cultivars (Yabukita and Benifuki).

Parts	Nicotine content (μg g^−1^ dry weight)					
	Yabukita			Benifuki		
New leaves	0.003	±	0.000 a	0.008	±	0.006 A
New stems	0.01	±	0.000 b	0.01	±	0.000 A
Mature leaves	0.009	±	0.001 b	0.009	±	0.000 A
Mature stems	0.012	±	0.001 b	0.01	±	0.001 A
White roots	0.011	±	0.000 b	0.029	±	0.003 B
Lignifying roots	0.013	±	0.004 b	0.024	±	0.003 B

Data are means ± S.D. (n = 3). Different letters indicate significant differences (*P* < 0.05, Tukey’s). Lowercase and uppercase characters indicate significant differences among organs of Yabukita and Benifuki, respectively.

Nicotine contents in flower buds and seeds of Yabukita are shown in Table 5. In flower buds, nicotine contents of filaments (0.041 μg g^−1^ DW) were higher than those of petals (0.031 μg g^−1^ DW) and anthers (0.033 μg g^−1^ DW). Seeds had nicotine contents (0.030 μg g^−1^ DW) similar to those of petals.

**Table 5:** Nicotine contents of flower buds and seeds of tea plants (Yabukita), cultured tea cells and B5 medium before and after 9 days cultivation, and leaves, stems and roots of tea seedlings cultured under aseptic conditions for 90 days.

Samples		Nicotine content
		(μg g^−1^ dry weight)
Flower buds^1)^	Petals	0.031
	Filaments	0.041
	Anthers	0.033
Seeds^1)^		0.030
B5 medium^2)^	Before use	n.q.
	After cultivation	n.q.
Tea cells^2)^		0.033 ± 0.019
Tea seedlings^1)^	Leaves	0.033
	Stems	0.044
	Roots	0.059

^1)^ Values are from a single replicate (*n* = 1).

^2)^ Values are means ± S.D. (*n* = 10). n.q.: not quantified [below the LOQ (0.005 μg g^−1^ dry weight)].

Nicotine contents of suspension-cultured tea cells and B5 medium before and after 9 days incubation are displayed in Table 5. Nicotine was detected in tea cells at a content of 0.031 μg g^−1^ DW, but was not found in B5 medium either before or after incubation.

Table 5 shows nicotine contents of leaves, stems and roots of tea seedlings grown under aseptic condition for 90 days. Nicotine was detected at a content of at least 0.033 μg g^−1^ DW in all plant organs, with the maximum level found in roots.

## Discussion

Among the tea samples collected from tea-producing localities in six Asian countries, we found detectable levels of nicotine in Japanese green tea (Table 1). This result reconfirms the presence of nicotine in Japanese green tea. With respect to maximum values among tea-producing regions, nicotine contents of organically grown black tea from Assam and Darjeeling were higher than those of Japanese green tea. In a study of 41 black, 14 oolong, 26 green and 6 white tea samples, conducted with LC-ESI-MS/MS using a modified QuEChERS method, Thräne et al. [17] observed that the highest nicotine contents (> 0.6 μg g^−1^ DW) were found in samples from India and China. They also reported that tea samples from Japan had contents below 0.19 μg g^−1^ DW. These results suggest that nicotine contents of tea leaves differ among producing regions and cultivars. In spite of the relatively small number of samples in the present study, we also observed that nicotine contents of black tea (0.024–0.694 μg g^−1^ DW) were significantly higher than those of green tea (0.011–0.40 μg g^−1^ DW) (Table 1). This trend is in line with average nicotine contents reported by EFSA 2011 [4] of 0.130 μg g^−1^ DW (0–0.873 μg g^−1^ DW) for black tea vs. 0.075 μg g^−1^ DW for green tea (0–0.440 μg g^−1^ DW)—although it should be noted that these samples did not include Japanese green tea. In tobacco plants, it is reported that nicotine synthesized in roots accumulates in vacuoles [18] and so also the flavonoids such as catechins are found in the vacuoles in tea [19]. Understanding the significance of these observed differences among producing regions and cultivars will require future investigation of cultivation environments, such as soil conditions and climate, and the introduction of additional cultivars. Thräne et al. [17] found that average nicotine contents seemed to increase with increasing degree of fermentation, although black teas with contents below a LOQ of 0.01 μg g^−1^ DW were also observed. In contrast, the results of Sheen [5] suggest that nicotine is reduced in tomato via enzymatic oxidation and other chemical reactions during processing. As is well known, tea is classified into three types based on the degree of fermentation: non-fermented (green tea), semi-fermented (oolong tea) and full-fermented (black tea). During the tea manufacturing process, leaves are typically heated by steaming (ca. 100°C), hot air exposure (65–115°C) or by placement on high-temperature pans (ca. 300°C) to diminish oxidative enzyme activities and for drying (S1 Fig). Nicotine contents in tea leaves would thus be expected to change during manufacturing. In this study, however, nicotine contents remained constant regardless of tea type (Sencha, pan-fired, oolong or black). This result suggests that heating and fermentation have no effect on nicotine contents in tea leaves. Similarly, the application of withering, a leaf-damaging process, during the manufacturing of oolong and black teas had no effect on nicotine contents.

A comparison of two cultivars revealed that nicotine contents in *C*. *sinensis* var. *sinensis* ‘Yabukita’ were lower than those of *C*. *sinensis* var. *sinensis* × var. *assamica* ‘Benifuki’ in new leaves subjected to tea manufacturing processes (Table 2), in new leaves of field-grown tea plants (Table 3) and in new leaves and roots of hydroponically grown tea plants (Table 4). These results suggest that nicotine contents of *C*. *sinensis* var. *assamica* are higher than those of var. *sinensis*. Because leaves of varieties *sinensis* and *assamica* are generally used to make green and black teas, respectively, this implies that a difference exists in nicotine accumulation among tea varieties. In samples of var. *assamica* from India, however, we detected nicotine contents as low as 0.100 μg g^−1^ DW, even though the highest value was also detected in an Indian tea sample (0.694 μg g^−1^ DW; Table 1). A wide range of nicotine contents in Indian black tea has also been observed in the above studies. Two possible explanations for these results can be proposed. One possibility is genetic variation in nicotine synthesis in tea plants; the other is environmental contamination due to various reasons—for example, from smokers (cigarette smoke or nicotine residues on fingers of harvesters) [20] or seepage into ground water from nearby cultivated tobacco plants [21]. Because nicotine was detected in all types of tea samples, the latter possibility seems unlikely but must still be considered.

New tea shoots (new leaves and stems) were harvested several times every year from spring to autumn. In general, plucking was carried out four times annually in India and two or three times in Japan. No clear seasonal trend, such as highest value, could be discerned in the nicotine content of Indian black teas collected from 14 tea estates (S4 Fig). In Japan, nicotine contents of new leaves and stems of field-grown tea plants were highest during second and third crop seasons (Table 3). In tobacco plants, damage to leaves increases nicotine contents, indicating that nicotine biosynthesis is affected in response to mechanical wounding [22–24]. Wang et al. [25] have also reported that excision of tobacco shoot apices causes dramatic increases in nicotine contents in leaves. Before beginning this study, we therefore assumed that wounding caused by plucking of tea new shoots might increase nicotine contents, thereby leading to higher levels in tea leaves during second and third crop seasons compared with the first season. Although this trend was observed in Japanese field-grown plants, no such pattern was uncovered in Indian black tea. In tobacco plants, nicotine biosynthesis is regulated by multiple biotic and abiotic stresses during the growing season, such as drought, heat and herbivore or insect damage [26, 27]. Because the type and degree of stress experienced under field conditions among tea-growing regions was variable, the individual seasonal fluctuation of nicotine content in new tea shoots differed between tea estates. The seasonal change in nicotine contents of new shoots observed in this study consequently suggests that nicotine biosynthesis and degradation is carried out in tea plants.

Leaves, stems and roots as well as seeds and flower buds also contained nicotine (Table 5). In field-grown tea plants, the annual average nicotine content was highest in roots (Table 3). Roots were also the organs with the highest contents of nicotine in tea plants grown hydroponically in a greenhouse and aseptic seedlings (Tables 4 and 5). In tobacco plants, nicotine is synthesized in roots [28], where it remains most abundant until topping [25]. On the basis of nicotine presence in other plant organs besides fruits, Sheen [5] concluded that tomato also biosynthesizes nicotine. Although no other reliable evidence is currently available, our data strongly suggest that nicotine is synthesized in the roots of tea plants. Similar to aseptic seedlings, suspension-cultured tea cells contained nicotine (0.033 μg g^−1^ DW) (Table 5). These cells were derived from anthers of Yabukita and had been sub-cultured weekly since 1994 in B5 medium under aseptic conditions. Nicotine was not detected in the B5 medium alone. Even when grown under these conditions, which virtually guaranteed the complete exclusion of nicotine contamination from exogenous sources, tea cells contained nicotine. These results strongly support that endogenous nicotine is present in tea plants.

In tobacco plants, nicotine is synthesized from putrescine, a polyamine produced either directly from ornithine in a reaction catalyzed by ornithine decarboxylase (EC 4.1.1.17) or indirectly from arginine in a reaction catalyzed by arginine decarboxylase (EC 4.1.1.17) [29, 30]. The first step in nicotine biosynthesis is the conversion of putrescine to N-methylputrescine, which is catalyzed by putrescine N-methyltransferase [31–35]. Although the final step of nicotine biosynthesis is still not known clearly, recently it was reported that an A622, a member of PIP family of NADPH-dependent reductases, and berberine bridge enzyme like proteins (BBLs) have these function [36]. Besides, the production of nicotine and the expression levels of genes involved in nicotine biosynthesis showed an increase after wounding and jasmonate treatment [37] mediated by the jasmonate signaling cascade, which is regulated defense responses against environmental stresses [38].Wang et al. [25] reported that nicotine content increases in tobacco plants harvest at the upper leaves during cultivation. In general, even in the cultivation of tea, the risk of infestation of insects and pathogenic attacks also increases in the summer season compared with the winter season. Indeed, our results represent that nicotine contents in tea differ in each region and season (Tables 1 and 3), especially their values tends to be high in summer season. In other words, environmental factors related to jasmonate signaling may affect nicotine biosynthesis in tea. The activity and gene expression of these enzymes have not been examined in tea plants. To provide further evidence of endogenous nicotine, molecular-level investigation of nicotine synthesis in tea plants is needed.

Compared with other plant species, e.g. solanaceous; < 100 μg kg^−1^ FW (fresh weight) on average [8, 10], it was confirmed that the content of nicotine contained in tea was of same level. Based on the EFSA report 2011 [4], intake of nicotine via a cup of tea (1.5 g leaf/150 ml) calculated from our results is 0.008 to 0.500 μg when calculated at a transition rate of 48% (median), and it is 0.017 to 1.041 μg when it is 100%, these were also reported to be of same level (≤ 1 μg day^−1^) as in solanaceous, and much lower than that via passive smoking (80 μg day^−1^) or smoking (number of cigarettes × 1 mg day^−1^) [10]. According to an EFSA report 2011 [4], tea is the major source of ingested nicotine among herbal infusions and spices, but its maximum contribution amounts to only 0.7% of acceptable daily intake. On this basis, EFSA proposed a MRL of 0.6 μg g^−1^ for tea. With the exception of a few samples, nicotine contents obtained in this study were lower than this value, which suggests that tea has no toxic effect when consumed as a beverage. In light of our data, we recommend future revision of this MRL, with careful consideration given to samples exceeding this value to avoid the risk of contamination.

## Conclusions

In this study, all purchased tea samples and samples of roots, stems, flower buds and seeds contained nicotine ranging from 0.003 to 0.694 μg g^−1^ DW. In addition, tea cells cultured in B5 medium without nicotine contained nicotine at a content of 0.033 μg g^−1^ DW. These results suggest that tea plants contain nicotine that is not derived from exogenous sources. In addition, nicotine contents in tea leaves changed seasonally and differed among cultivars. Furthermore, a difference in nicotine contents was observed among tea plant-growing regions. All these results strongly suggest that tea plants, similar to members of the Solanaceae, contain endogenous nicotine. Although low, the levels are higher than allowed MRLs. Despite these findings, the possibility of contamination of commercial tea by exogenous nicotine cannot be completely excluded.

## Supporting information

S1 Table Type, subtype, country, sampling year and number of tea samples used to measure nicotine in this study. (DOCX)

S1 Fig Manufacturing processes used to produce Sencha, pan-fired, oolong and black teas. (PDF)

S2 Fig New leaves, new stems, mature leaves, mature stems, lignifying roots and white roots of tea plants cultured hydroponically. The white bar corresponds to 10 cm.
(PDF)

S3 Fig **Flower buds and seeds (A), cultured tea cells (B) and aseptic tea seedlings (C) of Yabukita tea plants**.
(PDF)

S4 Fig Nicotine contents of tea samples collected during first, second, rain/monsoon and autumn seasons from individual tea estates (designated as A to S) in India. The dotted line indicates the residue standard for nicotine (0.01 μg g^−1^ dry weight).
(PDF)
