# Supplementary material for: Quantitative validation of nicotine production in tea (Camellia sinensis L.)
Source: PLoS One. 2018 Apr 9;13(4):e0195422. doi: 10.1371/journal.pone.0195422 (PMC5890992; doi:10.1371/journal.pone.0195422)
Supplement: S1 Table — (DOCX) [file pone.0195422.s001.docx]

| Type | Subtype | Country | | Sampling year | Number |
| --- | --- | --- | --- | --- | --- |
| Green tea | Sencha | Japan |  | 2014, 2015 | 31 |
|  | Panning | Taiwan |  | 2013 | 1 |
| Black tea |  | India | Assam | 2015 | 11 |
|  |  |  | Darjeeling | 2015 | 64 |
|  |  | China |  | 2012 | 1 |
|  |  | Taiwan |  | 2012 | 1 |
|  |  | Indonesia |  | 2015 | 1 |
|  |  | Vietnam |  | 2015 | 1 |
| Oolong tea |  | Taiwan |  | 2013 | 1 |
| Total |  |  |  |  | 112 |
