# Supplementary figures and images for: Quantitative validation of nicotine production in tea (Camellia sinensis L.)
Source: PLoS One. 2018 Apr 9;13(4):e0195422. doi: 10.1371/journal.pone.0195422 (PMC5890992; doi:10.1371/journal.pone.0195422)

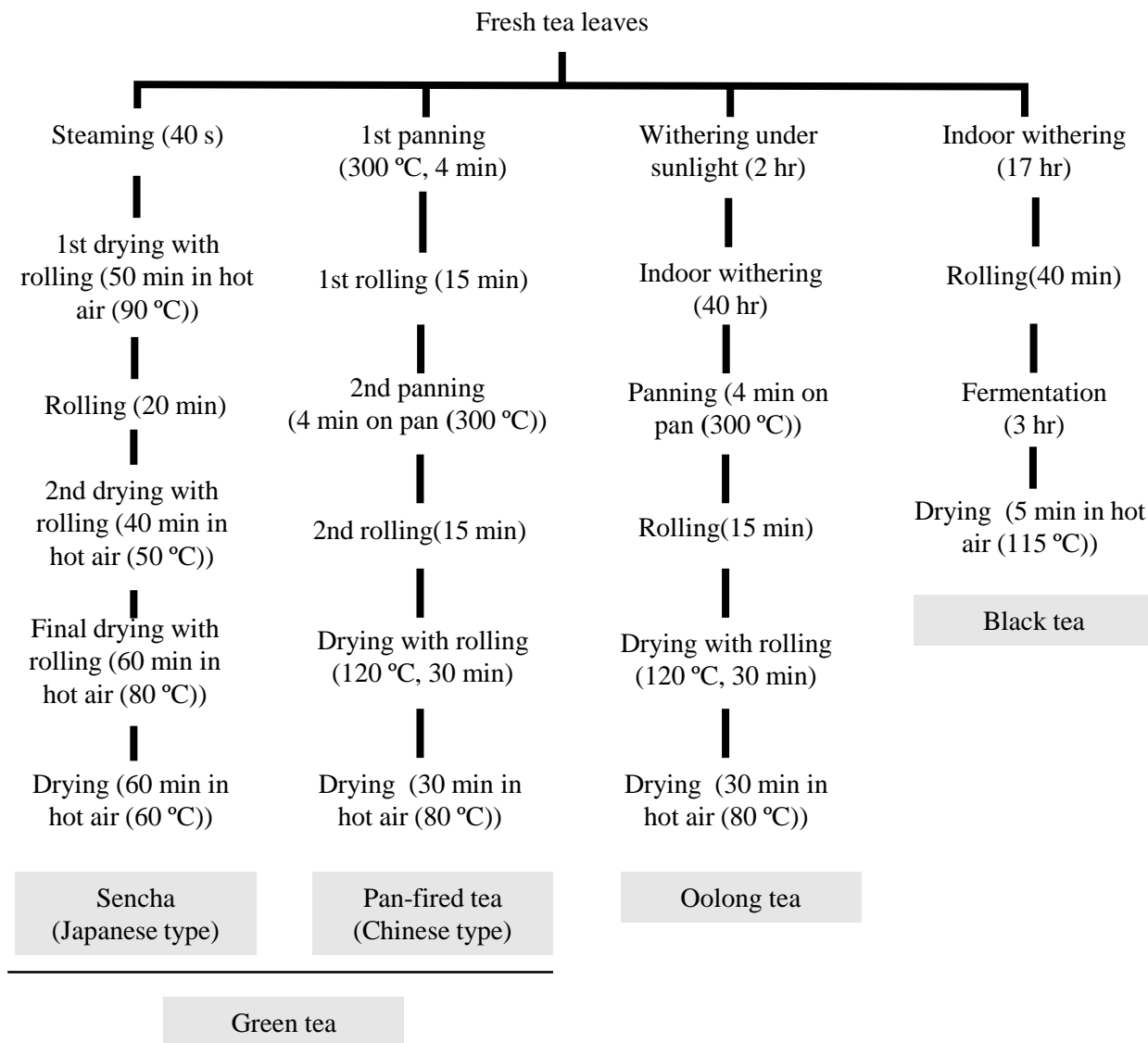

Supplement: S1 Fig — (PDF) [file pone.0195422.s002.pdf]

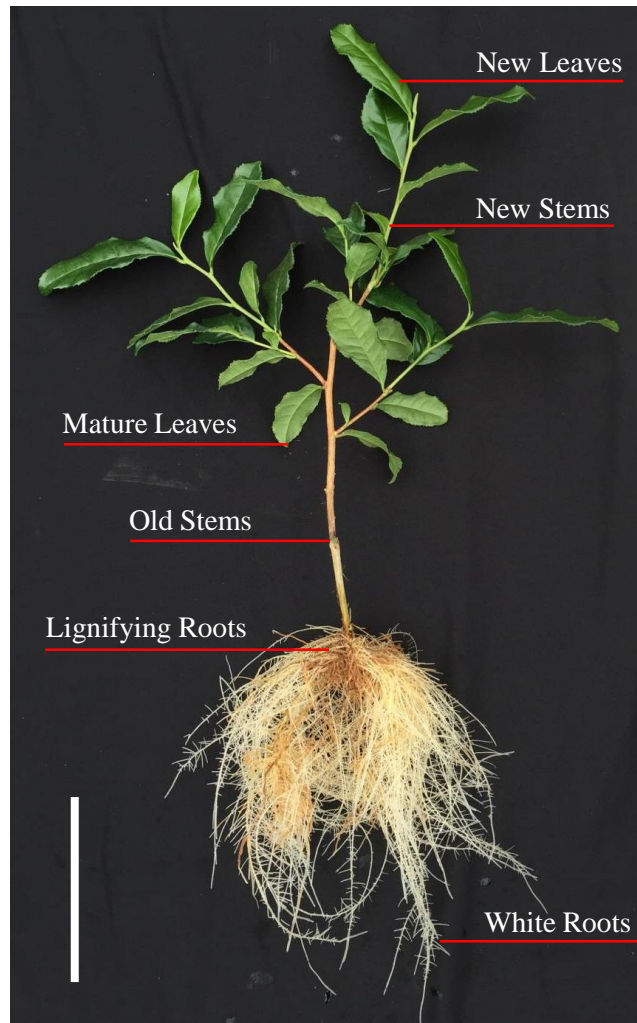

Supplement: S2 Fig — The white bar corresponds to 10 cm. (PDF) [file pone.0195422.s003.pdf]

(A)

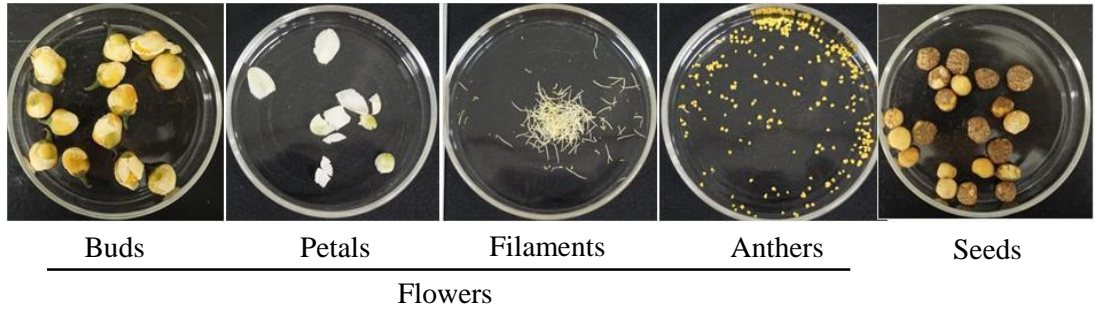

(B)

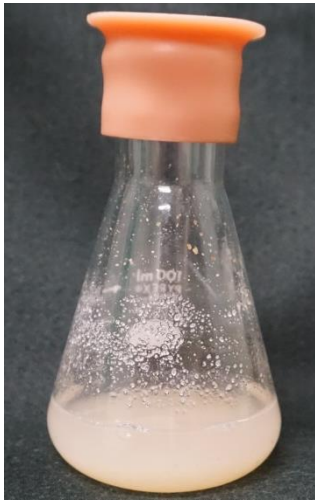

(C)

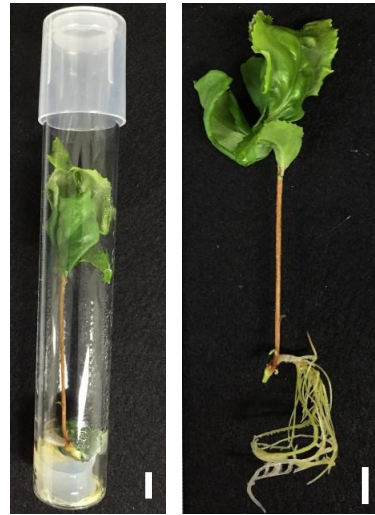

Supplement: S3 Fig — Flower buds and seeds (A), cultured tea cells (B) and aseptic tea seedlings (C) of Yabukita tea plants. (PDF) [file pone.0195422.s004.pdf]

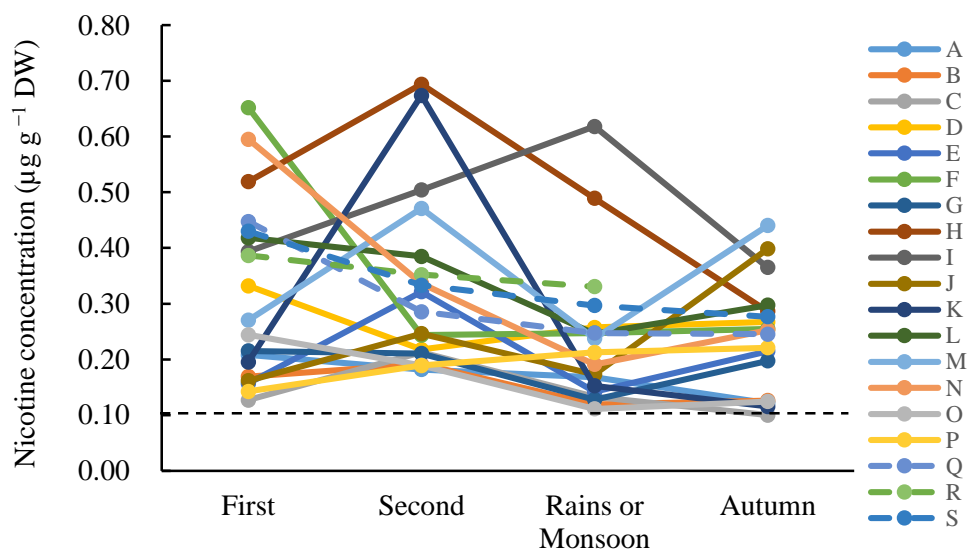

Supplement: S4 Fig — The dotted line indicates the residue standard for nicotine (0.01 μg g−1 dry weight). (PDF) [file pone.0195422.s005.pdf]
